# Alterations in Serum Adropin, Adiponectin, and Proinflammatory Cytokine Levels in OSAS

**DOI:** 10.1155/2020/2571283

**Published:** 2020-05-04

**Authors:** Hakan Celikhisar, Gulay Dasdemir Ilkhan

**Affiliations:** ^1^Izmir Metropolitan Municipality Hospital, Chest Diseases Clinic, Gaziler Caddesi No. 315, 35110 Izmir, Turkey; ^2^Okmeydanı Training and Research Hospital, Chest Diseases Clinic, Kaptan Pasa Mahallesi, Darulaceze Cad. No. 25, 34384 Istanbul, Turkey

## Abstract

**Objective:**

The present study was planned to examine the relationships between obstructive sleep apnea syndrome (OSAS) and the newly revealed adipokines adropin and adiponectin concentrations that display significant metabolic and cardiovascular functions and the levels of proinflammatory cytokine levels.

**Method:**

A total of 166 overweight and obese male patients with a body mass index (BMI) >27 kg/m^2^ were included in the study. Among study participants, 84 were recently diagnosed with OSAS by polysomnography with an apnea-hypopnea index (AHI) ≥5, and 82 were nonapneic with normal polysomnography (AHI<5) findings. The serum adropin and adiponectin levels of all cases were analyzed via the enzyme-linked immunosorbent assay method. Serum interleukin-1 (IL-1) beta and tumor necrotizing factor-alpha (TNF-alpha) levels were determined using Luminex cytokine multiplex analyses.

**Results:**

The mean age of the OSAS patients was 50.9 ± 5.7 years and BMI was 32.4 ± 6.0 kg/m^2^, and there was no statistically significant difference determined with the control group (49.3 ± 5.8 years and 30.6 ± 5, 6 kg/m^2^) (*p* > 0.05). There were no statistically significant differences between the OSAS and control groups concerning total cholesterol, triglyceride, low-density lipoprotein (LDL), high-density lipoprotein (HDL), and glucose levels. Adiponectin was lower in the OSAS group at a statistically significant level in comparison with the control group and was related at a statistically significant level to OSAS intensity. Adropin concentration was determined to be higher in the OSAS group at a statistically significant level in comparison with the control group.

**Conclusion:**

The results of our study suggest that increased adropin concentration may be an indicator of endothelium dysfunction in OSAS patients. Serum adropin and adiponectin levels may be new bioindicators used for diagnosis and risk assessment in OSAS patients.

## 1. Introduction

Cytokines are proteins synthesized as a response to inflammation primarily from macrophages, neutrophils, and endothelium cells [1]. Cytokines are classified into two groups according to their functions as proinflammatory and inflammatory. Cytokines such as interleukin-1 (IL-1), IL-6, and tumor necrotizing factor (TNF) are proinflammatory cytokines and play a role in putting forth the rapid immune response for the elimination of pathogens as well as the formation of inflammatory changes [2]. TNF has two different types known as alpha and beta. TNF-alpha is synthesized by lymphocytes, active macrophages, fibroblasts, and endothelium cells, causing inflammation, vascular thrombus development, tumor necrosis, synthesis of acute-phase reactants from the liver, cachexia, and fever, whereas TNF-beta is secreted from primary T lymphocytes and displays effects resembling those of TNF-alpha but only weaker [3, 4].

Fat tissue has biological activities related to energy metabolism, neuroendocrine function, and immune functions [4]. Adiponectin is an important adipocytokine in the adipose tissue that plays a role in the regulation of the differentiation of preadipocytes in addition to facilitating the synthesis of mature adipocytes. With a molecular weight of 30 kilodaltons, adiponectins are genetically coded proteins that are mostly found in the fat tissue [5]. Adiponectin expression is greater in the subcutaneous fat tissue than the visceral fat tissue [6]. It was determined in the clinical studies carried out that the adiponectin level is lower in obesity, diabetes mellitus, and coronary artery diseases [7]. Results obtained from similar studies indicate that the plasma adiponectin level is negatively related to insulin, leptin level, and visceral fat tissue [8]. Adropin, a recently defined peptide coded by the gene related with energy homeostasis, is strictly regulated by energy uptake and plays a role in cardiovascular functions, especially endothelium functions [9]. Hence, we assumed that the change in adropin concentrations is a proof of endothelium dysfunction especially due to the fact that hypoxia in patients with obstructive sleep apnea syndrome (OSAS) may be a reliable indicator for endothelium dysfunction within the context of OSA.

Obstructive sleep apnea syndrome results in alveolar ventilation and thus fluctuations in intrathoracic negative pressure due to the partial or complete repetition of upper respiratory tract collapse during sleep which is the reason for fragmented sleep architecture [9, 10]. It has been put forth that hypoxia and hypercapnia attacks and arousals during repeated apneas result in the overstimulation of the sympathetic nervous system and that, as a result, the inflammatory processes developed by peripheral vasoconstriction and triggered with hypoxia and endothelium dysfunction play a role in OSAS pathogenesis [11]. On the other hand, the intermittent hypoxemia related desaturation and reoxygenation pattern activates the oxidative stress system, thereby resulting in the formation of reactive oxygen products. This disrupts the synthesis and synthesis-degradation balance of oxidant-antioxidant metabolites [12]. Proofs acquired as a result of studies carried out during the last two decades support the necessity of considering OSAS-related sleep disorder as a low level of chronic inflammatory disease. Accordingly, it is considered that a portion of the morbid phenotypic development in OSAS can be explained causally by the underlying inflammatory processes that induce end-organ function disorder [11, 12].

The aim of the present study was to measure the tumor TNF-alpha, interleukin-1 beta (IL-1 beta), adiponectin, and adropin levels that play a role in systemic inflammation in OSAS patients for determining whether they are related to OSAS or not.

## 2. Materials and Methods

A total of 166 male patients subject to polysomnography (PSG) at the Izmir Eşrefpaşa Hospital Sleep Center during the dates of January 2018 and April 2019 were included in this prospective study. All cases were selected from among overweight and obese male patients with a body mass index (BMI) >27 kg/m^2^. Those with accompanying cardiovascular, metabolic, genetic, and rheumatic diseases, such as diabetes mellitus and coronary artery disease, along with those with alcohol–drug addiction were excluded from the study. The polysomnographic data of the cases, glucose, lipid profile, serum adropin, adiponectin, and cytokine levels in addition to sociodemographic data such as age, BMI, and smoking status were recorded after taking their informed consent.

Blood samples were drawn from the study group after a 12-hour fasting period with a 10 ml chilled syringe containing EDTA. Glucose, total cholesterol (TC), triglyceride, low-density lipoprotein (LDL), and high-density lipoprotein (HDL) cholesterol levels were measured from the drawn blood samples. The blood samples were taken for measuring serum adropin, adiponectin, and cytokine levels, were centrifuged at 2000 rpm for 10 minutes, and stored at −80°C until analyses. Serum adropin (Human AD [Adropin], ELISA Kit Synonyms: ENHO, Associated with Energy Homeostasis, Catalog No. E-EL-H5307; Elabscience, China) levels were analyzed via enzyme-linked immunosorbent assay (ELISA) method. The analytical detection range was 12.50–800 pg/mL for adropin. For the measurement of adiponectin levels, Adiponectin ELISA BioVendor (BioVendor lab. Medicine, Inc. Czech Rep.) kits were used. Serum, IL-1 beta, and tumor necrosis factor-alpha levels (TNF-alpha) were determined via Luminex cytokine multiplex analyses using a Fluorokine MAP kit (R&D Systems, Minneapolis, MN) and a Luminex device (Luminex, Austin, TX). Signed informed consent forms were obtained from all subjects.

## 3. Statistical Analysis

The purpose of the study was to determine whether adropin, adiponectin, interleukin-1 beta, TNF-alpha, triglyceride, cholesterol, HDL, LDL, and glucose levels of OSAS patients and the healthy control group differ at statistically significant levels or not and hence statistical methods were used for this purpose. *One-way variance analysis* was used for determining whether the adiponectin, triglyceride, cholesterol, and HDL and LDL values with respect to OSAS groups. The *Kruskal–Wallis H test* was used for determining whether the adropin and TNF-alpha values differ concerning OSAS groups. The value of *p* < 0.05 was used for statistical significance. Individuals included in the study were analyzed separately after being classified into two groups as OSAS and the control group. The *two independent samples t-test* was used for determining whether there are any statistically significant differences between the adiponectin, interleukin-1 beta, TNF-alpha, triglyceride, cholesterol, HDL and LDL value, and low-density lipoprotein between these two groups. The *Mann–Whitney U test* was used for determining whether the adropin and TNF-alpha values differ between these two groups or not.

## 4. Results

The study was carried out with a total of 166 male subjects aged between 45 and 59 years. In the control group of 82 subjects, OSAS was not observed (AHI<5). In the patient group of 84 subjects, mild OSAS was determined in 28 patients (33.3%), moderate OSAS in 26 (30.9%), and severe OSAS in 30 (35.7%). The mean age of all participants was 49.6 ± 6.5 years. Also, the mean age of the OSAS patients was 50.9 ± 5.7 years. The mean age of the control group was 49.3 ± 5.8, and there was no statistically significant difference between the two groups regarding the age (*p* > 0.05). The mean BMI of the OSAS and control groups was 32.4 ± 6.0 kg/m^2^ and 30.6 ± 5.6 kg/m^2^, respectively (*p* > 0.05). Of the OSAS patients, 23 (28%) were smokers, while there were 14 (18%) smokers in the control group; the difference between the two groups was not statistically significant regarding the smoking status (*p* > 0.05).  Table 1 presents the demographic and polysomnographic data of the patient and control groups.

The mean fasting blood glucose level was 102.5 ± 12.3 mg/dl for the patient group, whereas the fasting blood glucose was 98.7 ± 9.7 mg/dl for the control group. The triglyceride level of the patient group was 175.2 ± 108.2 mg/dl and that of the control group determined as 185.3 ± 89.2 mg/dl. The high-density lipoprotein (HDL) and low-density lipoprotein (LDL) levels of the patient group were determined as 45.3 ± 14.1 mg/dl and 132.8 ± 32.5 mg/dl, respectively, whereas the control group values were 46.2 ± 15.3 mg/dl and 126.4 ± 28.3 mg/dl, respectively. No statistically significant difference was observed between the OSAS patient group and the control group concerning fasting blood glucose, triglyceride, HDL, and LDL values (*p* > 0.05). Serum adiponectin levels were lower in the patient group (2.9 ± 3.6 *μ*g/dl) at a statistically significant level in comparison with the control group (6.1 ± 5.8 *μ*g/dl) (*p*=0.005). Serum adiponectin levels displayed a negative and statistically significant correlation with AHİ (*r* = −0.221; *p*=0.03).

**Figure 1:**
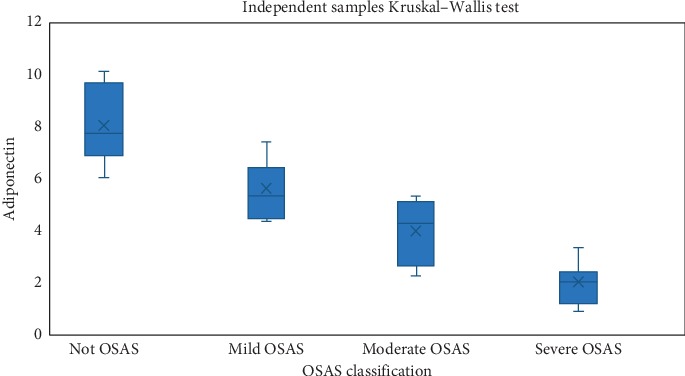
Distribution of adiponectin values according to OSAS classes.

A statistically significant difference could not be observed between the OSAS groups of the patients and their triglyceride, cholesterol, HDL, and LDL values (*p* > 0.005). In other words, the triglyceride, cholesterol, HDL, and LDL values of the patients do not change with an increase in OSAS severity as shown in Table 2. On the other hand, according to the Tukey test results carried out for determining the relationship between the OSAS groups with regard to adiponectin level, a statistically significant difference was determined between the control group (non-OSAS) and OSAS patient group (*p*=0.006). Accordingly, the adiponectin values of severe OSAS patients were determined to be lower in comparison with that of the non-OSAS patients. On the other hand, a statistically significant correlation was observed between the OSAS severity and adiponectin level as a result of the statistical evaluation carried out between the OSAS groups. The distribution of adiponectin levels according to OSAS classes is shown in Figure 1.

When the control group patients without OSAS were compared with mild, moderate, and severe OSAS groups regarding the adiponectin levels, the differences were all statistically significant (*p* values were 0.046, 0.024 and 0.010, respectively). Moreover, adiponectin levels were significantly lower in mild OSAS group than those of the moderate (*p*: 0.041) and severe (*p*: 0.023) OSAS groups and lower in moderate OSAS group than those of the severe OSAS group (*p*: 0.039).

**Figure 2:**
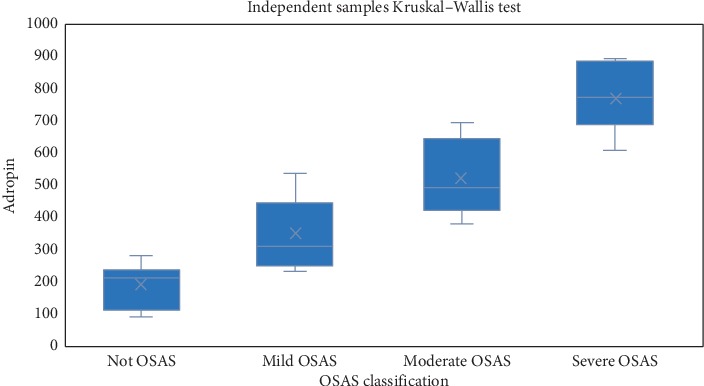
Distribution of adropin values according to OSAS classes.

When Tukey multiple comparison test results for adropin values were investigated, there was a statistically significant difference between the non-OSAS group and the moderate OSAS and severe OSAS groups concerning adropin values (*p* values were 0.006 and <0.001, respectively). Similarly, a statistically significant difference was observed between the adropin values of mild OSAS patients and severe OSAS patients (*p* = 0.026). In conclusion, it can be stated that adropin values increase regularly with increasing OSAS severity of the patients. The distribution of adiponectin according to OSAS classes is shown in Figure 2.

**Figure 3:**
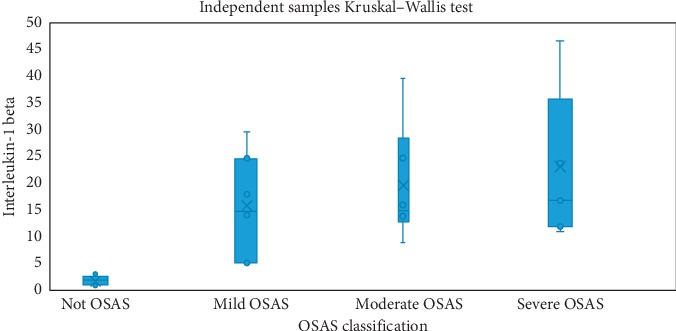
Distribution of the Interleukin-1 beta values according to OSAS classes.

**Figure 4:**
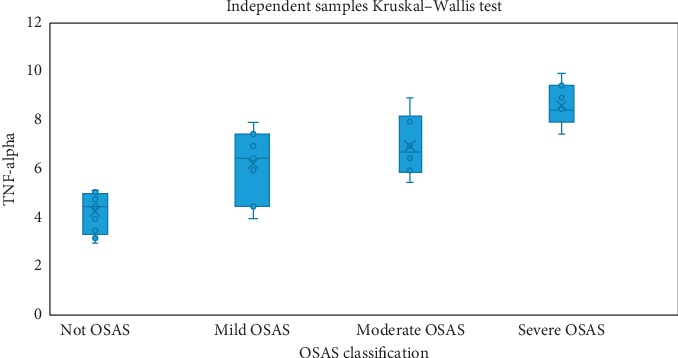
Distribution of TNF-alpha values according to OSAS classes.

Kruskal-Wallis H test was carried out for determining whether the IL-1 beta and tumor necrosis factor-alpha (TNF-alpha) values of the patients vary concerning OSAS classes or not. According to the results, a statistically significant difference was determined for both values between the OSAS classes (*p* < 0.001) (Table 3). Regarding IL-1 beta values, when the control group patients without OSAS were compared with mild, moderate, and severe OSAS groups, the differences were all statistically significant (*p* values were 0.016, 0.003 and <0.001, respectively). There was not any significant difference between OSAS groups concerning IL-1 beta levels. The distribution of IL-1 beta levels according to OSAS classes is shown in Figure 3. Similarly, in non-OSAS group, TNF-alpha levels were statistically significantly lower than that of the moderate or severe OSAS groups (*p* values were 0.024 and <0.001, respectively). The distribution of TNF-alpha levels according to OSAS classes is shown in Figure 4.

## 5. Discussion

In this study, a negative and statistically significant correlation was determined between the adiponectin levels and OSAS presence and severity. While the adropin level was determined to be higher at a statistically significant level in moderate or severe OSAS patients compared with healthy controls, a positive correlation was determined between the serum TNF-alpha, IL-1 beta levels, and OSAS presence.

Diseases such as OSAS that have courses with chronic intermittent hypoxia result in chronic systemic inflammation and oxidative stress. It has been put forth in a study carried out that the serum adiponectin levels relatively decrease in OSAS patients in comparison with simple snorers and it was argued that OSAS may result in a decrease in adiponectin levels [13]. Similarly, Kanbay et al. carried out a study in which a significant decrease was put forth in the adiponectin levels in the OSAS group, independent of obesity [14]. Masserini et al. carried out a study in which they compared 46 obese OSAS patients with the control group including 37 healthy individuals, as a result of which it was determined that the serum adiponectin levels decrease independent of the BMI and insulin resistance [15]. However, it was determined in another study that there was no statistically significant relationship between OSAS severity and serum adiponectin levels and it was suggested in the same study that the serum adiponectin levels oscillate due to the fat tissue independent of the presence of OSAS [16]. Nakagawa et al. reported a statistically significant decrease in adiponectin levels due to hypoxic stress [17]. The fact that a statistically significant difference could not be determined in our study between the age, gender, BMI, number of smokers, and accompanying diseases for the patient and control groups indicates the presence of a direct relationship between the decrease in serum adiponectin levels and OSAS severity. The statistically significant correlation between serum adiponectin levels and OSAS classification and AHI supports this result. In other words, as the AHI score that detects the presence of the diseases and determines the OSAS classification increases in our study, the serum adiponectin levels decrease at a statistically significant level.

The reduced secretion of adipokines induced by the activation of the sympathetic system may also trigger the cardiovascular results of obstructive sleep apnea syndrome. The relationship between OSAS severity and coronary artery disease risk may be due to the adverse impacts of hypoxia on endothelium functions, sympathetic activity, and increase in the inflammatory response and classical risk factors (obesity, HT, insulin resistance, and hyperlipidemia). Jun et al. carried out a study as a result of which it was put forth that intermittent hypoxia decreases triglyceride clearance [18]. The results obtained from similar studies show that chronic intermittent hypoxia may result in pathological changes in cholesterol and triglyceride metabolism resulting in increased lipolysis and increased lipid biosynthesis [18, 19]. This results in the synthesis from the adipose tissue at the molecular level of molecules that contribute to the protection of tissue homeostasis such as adiponectin. The process continues with the decrease in the secretion of anti-inflammatory adipokines such as adiponectin and the secretion from the adipose tissue of proinflammatory cytokines (such as IL-1, IL-6, and TNF-alpha) [19].

Adiponectin is found mainly in three forms in the human plasma: trimer, hexamer, and high molecular weight forms. High molecular weight (HMW) adiponectin is the active form of adiponectin and comprises the majority of intracellular adiponectin. HMW adiponectin plays a more active role in glucose and lipid metabolism in comparison with total adiponectin. However, even though the serum adiponectin levels have been measured in our study, the molecular forms have not been evaluated. This may be a limitation of the study. It still leads us to think that the total adiponectin levels decrease in OSAS patients in comparison with the controls and that the OSAS patients may have lower HMW adiponectin levels. It is known that serum adiponectin levels are affected by age and that they display a positive relationship with age [19]. There was no statistically significant difference between patients and control groups in our study. Moreover, all participants in our study were males to exclude the impact of gender on adiponectin levels; thereby, the impact of changes in adiponectin levels due to physiological and hormonal factors was prevented.

The activation of the TNF-alpha system has been put forth clearly in chronic obstructive lung disease characterized by chronic hypoxemia; however, the impact of hypoxemic conditions on cytokine generation in OSAS is less known. It is possible in OSAS patients that proinflammatory cytokine production will be increased by short, but repeated hypoxemic attacks and reactive oxygen products through mononuclear cells. TNF-alpha is strongly correlated with lipolysis and this cytokine causes insulin resistance. A statistically significant increase was observed in the adropin, IL-1 beta, and TNF-alpha levels of OSAS patients in this study.

Even though experimental proofs put forth that being subject to hypoxia may trigger insulin resistance, the results on the relationship between OSAS and glucose level and insulin resistance are contradictory. Some studies have put forth that the plasma glucose levels are higher at a statistically significant level in OSAS patients in comparison with the controls [20, 21]. However, Chung et al. [22] reported that insulin resistance was related to obesity itself rather than OSAS severity or nocturnal hypoxemia-related variables in their study. It was determined in our study that there are no statistically significant differences between the control group and the OSAS group concerning BMI; and also there was no statistically significant correlation between the fasting glucose levels of the controls and OSAS patients. Similarly, a statistically significant difference was not observed between the total cholesterol, HDL, LDL, and triglyceride values of the OSAS and control group. Increased lipid peroxidation has been reported for OSAS patients [23]. Obesity and thus increased fat tissue are indicated among the predisposing factors for OSAS. The reason why a statistically significant relationship could not be determined in our study between the patient and control groups concerning lipid profile may be explained by the fact that the BMI values do not display a statistically significant difference between the control and patient groups. Similar to our results, in a study of Ciftci et al. on obese patients classified into two groups, OSAS and non-OSAS, it was shown that the serum IL-1 beta and TNF-alpha levels are not correlated with AHI and BMI in the non-OSAS group but that the levels of these molecules are correlated with AHI but not BMI in OSAS patients. As a result, they determined that inflammation indicators increase in OSAS independent of BMI [24]. In a meta-analysis, Li et al. reported that circulating TNF-alpha levels were significantly higher in OSAS patients than that of the controls, and this difference became more pronounced with an increased severity of OSAS [25].

It has been determined in another study by Popko et al. examining the relationship between OSAS severity and inflammation indicators that plasma IL-1 beta levels increase with apnea intensity measured by AHI [26]. It was observed in our study that adropin and cytokines increase in OSAS and that the adiponectin serum levels decrease. Previous studies have put forth that obstructive sleep apnea is accompanied by increased adropin levels, but only when accompanied by endothelium function disorder. Hence, the study results confirm that adropin concentration is a reliable indicator of endothelium dysfunction in OSAS patients. It will be beneficial to know the exact molecular mechanisms of adropin impact in special tissues, certain diseases, or physiological states.

## 6. Conclusion

Serum adiponectin and adropin levels may be new bioindicators for determining the risk levels in OSAS patients. It would be possible to use these molecules for risk determination or even diagnosis and treatment as our knowledge increases on their roles in diseases with inflammation and certain physiological cases as well as their impact mechanisms on certain tissues.

## Figures and Tables

**Table 1:** Comparison of demographic and polysomnographic characteristics in patients with obstructive sleep apnea syndrome and in the control group.

	OSAS patient group			Control group			
	*n*	%	mean ± SD	*n*	%	mean ± SD	*p*
Age (years)	**84**		50.9 ± 5.7			49.3 ± 5.8	>0.05
BMI (kg/m^2^)			32.4 ± 6.0			30.6 ± 5.6	>0.05
Smoking	23	28		14	18		>0.05
AHI			27.4 ± 18.6			1.8 ± 1.4	

OSAS: obstructive sleep apnea syndrome; BMI: body mass index; AHI: apnea hypopnea index.

**Table 2:** One-way variance analysis results for the adiponectin, adropin, IL-1 beta, TNF-alpha, triglyceride, cholesterol, HDL, LDL, and glucose values of OSAS groups.

Variable	OSAS class	*n*	Mean	SD	Test value	*p*
Adiponectin	Non-OSAS	82	5.40	2.78	4.889	**0.006** ^*∗∗*^
	Mild OSAS	28	3.42	1.57		
	Moderate OSAS	26	2.80	1.92		
	Severe OSAS	30	2.05	1.01		

Adropin	Non-OSAS	82	188,88	30,55	424,665	**<0.001** ^*∗∗*^
	Mild OSAS	28	354,28	61,74		
	Moderate OSAS	26	523,33	48,98		
	Severe OSAS	30	774,28	36,54		

IL-1 beta	Non-OSAS	82	1.70	0.21	31.972	**<0.001** ^*∗∗*^
	Mild OSAS	28	11.80	7.04		
	Moderate OSAS	26	17.20	5.96		
	Severe OSAS	30	23.74	2.98		

TNF-alpha	Non-OSAS	82	3.80	1.08	19.440	**<0.001** ^*∗∗*^
	Mild OSAS	28	5.90	3.04		
	Moderate OSAS	26	7.30	2.45		
	Severe OSAS	30	8.60	2.35		

Triglyceride	Non-OSAS	82	189.80	57.05	0.166	0.919
	Mild OSAS	28	181.70	55.15		
	Moderate OSAS	26	185.41	28.88		
	Severe OSAS	30	199.40	24.38		

Cholesterol	Non-OSAS	82	217.00	28.51	0.645	0.591
	Mild OSAS	28	201.40	29.10		
	Moderate OSAS	26	208.30	30.00		
	Severe OSAS	30	215.77	17.70		

HDL	Non-OSAS	82	43.79	10.50	0.170	0.916
	Mild OSAS	28	41.70	18.90		
	Moderate OSAS	26	43.40	10.01		
	Severe OSAS	30	46.35	11.16		

LDL	Non-OSAS	82	130.01	24.83	0.126	0.944
	Mild OSAS	28	128.10	26.80		
	Moderate OSAS	26	123.61	12.67		
	Severe OSAS	30	128.10	17.45		

Glucose	Non-OSAS	82	100.72	11.21	0.126	0.975
	Mild OSAS	28	98.13	13.83		
	Moderate OSAS	26	95.61	12.67		
	Severe OSAS	30	102.17	14.25		

OSAS: obstructive sleep apnea syndrome; Ort: average; SS: standard deviation; TNF-alpha: tumor necrosis factor-alpha; HDL: high-density lipoprotein; LDL: low-density lipoprotein.^*∗∗*^ Statistical significance.

**Table 3:** Kruskal–Wallis *H* test results carried out for determining the difference between the OSAS classes and IL-1 beta and TNF-alpha values.

Variable	OSAS class	*N*	Mean	SD	Kruskal–Wallis *H* test	
					Test	*p*
IL-1 beta	Non-OSAS	82	1.70	0.21	31.972	**<0.001** ^*∗*^
	Mild OSAS	28	11.80	7.04		
	Moderate OSAS	26	17.20	5.96		
	Severe OSAS	30	23.74	2.98		

TNF-alpha	Non-OSAS	82	3.80	1.08	19.440	**<0.001** ^*∗*^
	Mild OSAS	28	5.90	3.04		
	Moderate OSAS	26	7.30	2.45		
	Severe OSAS	30	8.60	2.35		

SD: standard deviation; OSAS: obstructive sleep apnea syndrome.^*∗*^Statistical significance.

## Data Availability

The data used to support the findings of this study are available from the corresponding author upon request.
